# Electronic Cigarettes Use and Intention to Cigarette Smoking among Never-Smoking Adolescents and Young Adults: A Meta-Analysis

**DOI:** 10.3390/ijerph13050465

**Published:** 2016-05-03

**Authors:** Jieming Zhong, Shuangshuang Cao, Weiwei Gong, Fangrong Fei, Meng Wang

**Affiliations:** 1 Zhejiang Provincial Center for Disease Control and Prevention, 3399 Binsheng Road, Hangzhou 310051, China; jmzhong@cdc.zj.cn (J.Z.); wwgong@cdc.zj.cn (W.G.); frfei@cdc.zj.cn (F.F.); 2 Yidu Central Hospital of Weifang, 4138 Linglong Road, Qingzhou 262500, China; caoss1234@163.com

**Keywords:** electronic cigarette, smoking intention, meta-analysis

## Abstract

Electronic cigarettes (e-cigarettes) use is becoming increasingly common, especially among adolescents and young adults, and there is little evidence on the impact of e-cigarettes use on never-smokers. With a meta-analysis method, we explore the association between e-cigarettes use and smoking intention that predicts future cigarette smoking. Studies were identified by searching three databases up to January 2016. The meta-analysis results were presented as pooled odds ratio (OR) with 95% confidence interval (CI) calculated by a fixed-effects model. A total of six studies (91,051 participants, including 1452 with ever e-cigarettes use) were included in this meta-analysis study. We found that never-smoking adolescents and young adults who used e-cigarettes have more than 2 times increased odds of intention to cigarette smoking (OR = 2.21, 95% CI: 1.86–2.61) compared to those who never used, with low evidence of between-study heterogeneity (*p* = 0.28, I^2^ = 20.1%). Among never-smoking adolescents and young adults, e-cigarettes use was associated with increased smoking intention.

## 1. Introduction

Although reductions in the smoking prevalence were observed at global level since 1980, the tobacco pandemic remains a threat to the health of the world’s population [1]. As the second most important risk factor for global disease burden, tobacco use accounted for 6.1 million deaths and 143.5 million disability-adjusted life-years (DALYs) across the world in 2013 [2]. Currently, nations are striving to curb tobacco use and reduce its harm. Considering that the majority of smokers begin to smoke during their adolescence [3], preventing youth initiation and transition to established smoking are critical public health issues that deserve more attention.

Smoking intention, defined as the lack of a firm commitment not to smoke among never-smokers, is strongly predictive of future established smoking [4,5,6]. A growing body of literature has identified varying factors associated with smoking intention, such as parental or peer smoking, exposure to secondhand smoke inside or outside the home, pro-tobacco advertising, and school connectedness [7,8,9]. However, additional studies are warranted in this direction with the advent of electronic cigarettes (e-cigarettes).

E-cigarettes are battery-powered nicotine-delivery devices that mimic conventional cigarettes by vaporizing a liquid mixture consisting of propylene glycol, glycerin, flavorings, nicotine, and other chemicals. Since invented in 2003, e-cigarettes have been hotly debated regarding the safety and efficacy for smoking cessation [10,11,12,13,14]. While arguments between both sides of advocates and critics persist, e-cigarettes use has rapidly increased globally [15], especially among youth [16,17]. Moreover, given the majority of users among youth are never-smokers [18,19], whether youth use of e-cigarettes may serve as a gateway to cigarette smoking has been discussed in previous studies [20,21]. However, up to date, the effect of youth e-cigarettes use on subsequent cigarette smoking remains unclear. In the present study, we use meta-analysis method to explore the association between e-cigarettes use and smoking intention among adolescents and young adults to contribute to the much-needed evidence.

## 2. Materials and Methods

### 2.1. Literature and Search Strategy

Epidemiological studies on the association between e-cigarettes use and smoking intention were searched through three databases (PubMed, Springer Link, and Elsevier) from 2003 to January 2016. Smoking intention was defined as the lack of a firm commitment not to smoke among never-smokers, with the answer yes to one or two questions derived from previous studies [4,5,6]: “Do you think you will smoke a cigarette in the next year (or two years)?” and “If one of your best friends were to offer you a cigarette, would you smoke it?” Detailed definitions were shown in Table 1. The main search terms included “electronic cigarette”, “e-cigarette”, “electronic nicotine delivery systems”, “vaping”, “vaper”, “vapor”, “smoking intention”, “susceptibility to smoke”, “openness to smoke”, and “willingness to smoke”. Reference lists of retrieved literature were also screened.

The present study was carried out following the Meta-analysis of Observational Studies in Epidemiology (MOOSE) guidelines [28].

### 2.2. Inclusion Criteria and Data Extraction

Selected studies in this meta-analysis met the following criteria: (1) reporting the association between e-cigarettes use and smoking intention; (2) providing the effect value with 95% confidence interval (CI) or data to calculate these; (3) the study population must be never-smokers. Two authors independently assessed the eligibility of studies and extracted information from each eligible study. The information included (1) name of the first author; (2) year of publication; (3) participants and sample size; (4) data source and location; (5) study type; (6) measures definition; (7) variables adjusted.

### 2.3. Statistical Analysis

Heterogeneity between studies was assessed using a Q-test and the I^2^ statistic [29]. For the Q-test, *p* value < 0.05 was considered statistically significant. The low, moderate, and high degrees of heterogeneity correspond to I^2^ values of 25%, 50%, and 75%, respectively. If there was significant heterogeneity, a random-effects model would be used to assign the weight of each study according to the DerSimonian-Laird method [30]. If there was evidence of no heterogeneity, we used a fixed-effects model with effect estimates that were given equal weight to the inverse variance of the study. To test robustness of the present meta-analysis result, a sensitivity analysis was performed with excluding outliers. Publication bias was assessed by Egger’s regression asymmetry test (*p* value < 0.05 was considered statistically significant). All the statistical analyses were conducted with STATA Version 11 software (StataCorp LP, College Station, TX, USA).

## 3. Results

### 3.1. Study Characteristics

**Figure 1:**
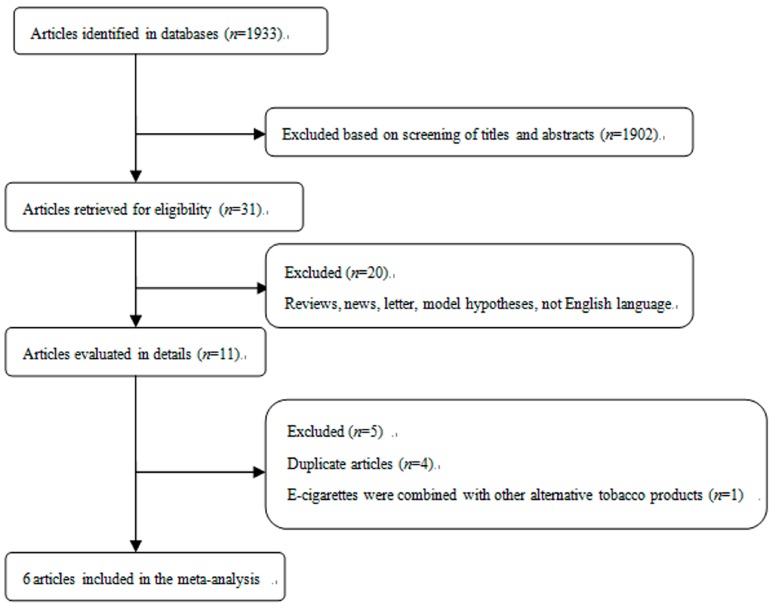
Selection of studies for inclusion in meta-analysis.

The process of study selection for this meta-analysis is shown in Figure 1. 20 articles were excluded from 31 potentially eligible studies because they were reviews, news, studies on model hypotheses, and/or published without English language. Additionally, it should be noted that four duplicate articles and one article that combined e-cigarettes with other alternative tobacco products were also excluded. The detailed information of studies was shown in Table 1. Briefly, a total of six studies were included in this meta-analysis of e-cigarettes use and smoking intention [22,23,24,25,26,27]. Among them, four studies were from the USA, one from China, and one from the UK. All the included studies reported the final estimates with adjustment for specified confounders.

### 3.2. Meta-Analysis of Association between E-Cigarettes Use and Smoking Intention

**Figure 2:**
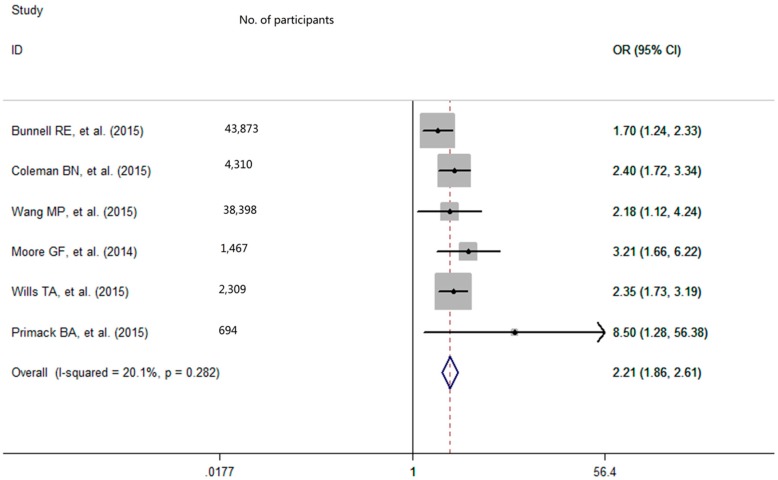
Effect of e-cigarettes use on smoking intention among never-smoking adolescents and young adults. OR refers to odds ratio; CI refers to confidence interval.

With low degree of heterogeneity (*p* = 0.28, I^2^ = 20.1%), a fixed-effects model was used to calculate the pooled odds ratio (OR) with 95% confidence interval (CI) for e-cigarettes use. The pooled analysis showed that among never-smoking adolescents and young adults, individuals who used e-cigarettes had a greater smoking intention in the future (OR = 2.21, 95% CI: 1.86–2.61; Figure 2). After excluding the outlier, the sensitivity analysis result showed that the pooled OR was 2.46 (95% CI = 2.01–3.01) and there was no significant study heterogeneity (*p* = 0.64, I^2^ = 0%).

### 3.3. Publication Bias

As for the publication bias, the Egger’s regression asymmetry test did not give a statistically significant result (*p* = 0.15).

## 4. Discussion

The present study uses meta-analysis method to provide a summary estimate of the effect of e-cigarettes use on smoking intention among never-smoking adolescents and young adults. With low evidence of between-study heterogeneity, findings from our meta-analysis confirm that never-smoking adolescents and young adults who used e-cigarettes have more than two times increased odds of intention to cigarette smoking (OR = 2.21, 95% CI: 1.86–2.61) in the future. Currently, there is considerable controversy about the health effects of e-cigarettes and one potential risk that e-cigarettes may become a new gateway to cigarette use among never-smokers has been identified as a concern by public health professionals [31,32]. As smoking intention is a strong predictor of future established smoking [4,5,6], our findings, in a way, add some tentative support for the probability that e-cigarettes use encourages the cigarette smoking initiation among never-smoking adolescents and young adults.

Although mechanisms accounting for the effect of e-cigarettes use on cigarette smoking initiation are complicated and still remain unclear, it is plausible that the nicotine exposure from e-cigarettes may play an important role in the “gateway effect”. As a nicotine-delivery product, e-cigarettes may serve as a “nicotine starter” [27]. Adolescents, who have developing brains, are especially sensitive to nicotine exposure [12], which can create nicotine dependence and lead youth to use tobacco products [33,34]. Currently, new-generation e-cigarettes have evolved to be more efficient and nicotine delivery from some devices may approach or even exceed that of a tobacco cigarette [35,36]. In theory, the “gateway effect” would be more apparent in the future. Additionally, secondhand tobacco smoke (SHS) is an important contributor to nicotine and may potentiate the smoking initiation. A recent systematic review reveals positive associations between smoking initiation among nonsmokers and SHS and nicotine dependence, [37]. E-cigarettes, with some of mainstream vapor exhaled, the secondhand exposure to bystanders is no doubt inevitable and has been identified as a source of nicotine [38,39]. Hence, we posit that the same process may promote youth who used e-cigarettes to cigarette smoking initiation. However, given that SHS is a significant public health problem among never-smoking adolescents [40], the effect of nicotine from e-cigarettes vapor on smoking initiation may be confounded by SHS inevitably. Except for the biological mechanism of nicotine, psychological mechanism is also possible. Studies have shown that the greater possibility of e-cigarette use is associated with increased exposure to parental or peer smoking, which may foster smoking susceptibility [40,41,42], suggesting that e-cigarette use is likely to be a proxy for influences and pressures from family and peer.

The results from our meta-analysis study are subject to several limitations. Firstly, our literature searching was conducted through only three databases and the possible bias was inevitable. Secondly, given that most of the included studies are cross-sectional studies in the current meta-analysis, we cannot infer whether the association between e-cigarettes use and cigarette smoking initiation is causal or not, and as such, more prospective studies are warranted. Thirdly, the data was self-reported and susceptible to misreporting. Fourthly, due to a limited number of participants who ever used e-cigarettes, the observed association between ever e-cigarettes use and smoking intention should be interpreted with caution.

## 5. Conclusions

From a public health perspective, our findings that e-cigarettes use by never-smoking adolescents and young adults is associated with cigarette smoking intention have important implications for the debates on the benefits and risks of e-cigarettes. In order to reduce the smoking intentions of youth and prevent them from initiating the first cigarette, we propose that prevention efforts around e-cigarettes restrictions should be enhanced.

## Figures and Tables

**Table 1:** Information of the studies included in the meta-analysis.

Study (year)	Source	Participants (No. of Ever e-Cigarettes Users/Participants)	Location	Design	Measures	Variables Adjusted
Bunnell RE, *et al.* (2015) [22]	2011–2013 National Youth Tobacco Survey (NYTS)	Students in grades 6–12 (541/43,873)	USA	Cross-sectional	**2011–2013 National Youth Tobacco Survey (NYTS)**	Sex, race/ethnicity, school level, number of distinct sources of pro-tobacco advertisement exposure, presence of a tobacco user in the household, and survey year
					Smoking intentions: “Do you think you will smoke a cigarette in the next year?” and “If one of your best friends were to offer you a cigarette, would you smoke it?” Response options included: “definitely yes”, “probably yes”, “probably not”, and “definitely not”. Those who responded “definitely not” to both intentions questions were classified as not having intentions; otherwise, respondents were classified as having intentions. Ever electronic cigarettes use: students who selected “Electronic Cigarettes or E-cigarettes, such as Ruyan or NJOY” to the question “Which of the following products have you ever tried, even just one time?” were considered ever e-cigarette users.	
Coleman BN, *et al.* (2015) [23]	2012–2013 National Adult Tobacco Survey (NATS)	Young adults aged 18–29 years (341/4310)	USA	Cross-sectional	**2012–2013 National Adult Tobacco Survey (NATS)**	Sex, age group, race/ethnicity, educational attainment, US Census region, ever use of smokeless tobacco, ever use of hookah, ever use of cigars, and ever experimentation with cigarettes
					Openness to smoking: “Do you think you will smoke a cigarette soon?” and “Do you think you will smoke a cigarette in the next year?” Response options were: “Definitely yes”, “Probably yes”, “Probably not”, and “Definitely not”. A binary composite variable was created, and those who responded with any response option other than a firm intention not to smoke (“Definitely not”) were categorized as being open to smoking cigarettes and, therefore, considered at risk for future smoking. Ever electronic cigarettes use: Those who had heard of electronic cigarettes or e-cigarettes and answered “yes” to the question “Have you ever used an electronic cigarette, even just one time in your entire life?”	
Wang MP, *et al.* (2015) [24]	2012–2013 Youth Smoking Survey	Secondary 1 to 6 students with mean age of 14.6 years (59/38,398)	Hong Kong	Cross-sectional	**2012–2013 Youth Smoking Survey**	Sex, age, perceived family affluence, peer smoking, parental smoking, and school clustering effect
					Smoking intentions: Students reported whether they would smoke in the next 12 months, and when cigarettes were offered by one of their good friends in two separate items each with four response options of “definitely not”, “probably not”, “probably yes”, and “definitely yes”. Those who chose “definitely not” for both questions were regarded as having no intention to smoke and otherwise as having an intention to smoke. Electronic cigarette use: Students who reported e-cigarettes use in the past 30 days, even one puff.	
Moore GF, *et al.* (2014) [25]	2014 Child exposure to Environmental Tobacco Smoke (CHETS) Wales 2	10–11 year-old children (77/1467)	Wales	Cross-sectional	**2014 Child exposure to Environmental Tobacco Smoke (CHETS) Wales 2**	Parents smoke/use e-cigarettes, friends smoking, sex, family affluence Scale (FAS)
					Smoking intentions: Future intentions were measured by the question ‘Do you think you will smoke in 2 years’ time?’, with response options of ‘definitely yes’, ‘probably yes’, ‘maybe or maybe not’, ‘probably no’, and ‘definitely no’. Electronic cigarettes use: Children were asked ‘Have you ever used an e-cigarette?’ with response options of ‘no’, ‘yes, once’, or ‘yes, more than once’. Children were classified as having used an e-cigarette if they responded ‘yes, once’ or ‘yes, more than once’. E-cigarettes were defined as electronic versions of cigarettes which do not give off smoke.	
Wills TA, *et al.* (2015) [26]	Survey at four public and two private high schools	High school students with mean age of 14.7 years (418/2309)	Hawaii	Cross-sectional	**Survey at four public and two private high schools in Hawaii**	Gender, ethnicity, family structure, parental education, parental support, parental monitoring, parent-adolescent conflict, academic competence, social competence, sensation seeking, rebelliousness, smoking expectancies, prototypes of smokers, peer smoker affiliation
					Willingness to smoking: “Suppose you were with a group of friends and there were some cigarettes you could have if you wanted. How willing would you be to ___:” The items were “Take one puff”, “Smoke a whole cigarette”, and “Take some cigarettes to try later”. Responses were on four-point scales with response points: Not At All Willing (0); A Little Willing (1); Somewhat Willing (2); and Very Willing (3). A composite score for willingness to smoke was the sum of the three items (α = 0.91). Electronic cigarettes use: The item on e-cigarettes was introduced with the stem: “Which of the following is most true for you about smoking electronic cigarettes (E-cigarettes, Volcanos)? (check one)”. Responses were on a seven-point scale with anchor points Never Smoked an E-cigarette in My Life to Usually Smoke E-cigarettes Every Day.	
Primack BA, *et al.* (2015) [27]	Second and third waves of the United States-based Dartmouth Media, Advertising, and Health Study	Adolescents and young adults aged 16–26 years (16/694)	USA	Longitudinal cohort	**Second and third waves of the United States-based Dartmouth Media, Advertising, and Health Study**	Sex, age, race/ethnicity, maternal education level, sensation-seeking tendency, parental smoking, close friends smoking
					Smoking intentions: “If one of your friends offered you a cigarette, “Would you try it?” and “Do you think you will smoke a cigarette sometime in the next year?” Responses included “definitely yes”, “probably yes”, “probably no”, and “definitely no”. Those who responded “definitely no” to both measures are considered Non susceptible nonsmokers (NSNS), whereas those who cannot rule out smoking are defined as susceptible. Electronic cigarettes use: whether participants had ever used an e-cigarette at baseline? (yes and no).	

## Abbreviations

E-cigarettes	Electronic cigarettes
OR	Odds ratio
RR	Relative risk
CI	Confidence interval
DALYs	Disability-adjusted life-years
SHS	Second hand tobacco smoke

## References

[1] Ng M., Freeman M.K., Fleming T.D., Robinson M., Dwyer-Lindgren L., Thomson B., Wollum A., Sanman E., Wulf S., Lopez A.D. (2014). Smoking prevalence and cigarette consumption in 187 countries, 1980–2012. JAMA.

[2] Forouzanfar M.H., Alexander L., Anderson H.R., Bachman V.F., Biryukov S., Brauer M., Burnett R., Casey D., Coates M.M., GBD 2013 Risk Factors Collaborators (2015). Global, regional, and national comparative risk assessment of 79 behavioural, environmental and occupational, and metabolic risks or clusters of risks in 188 countries, 1990–2013: A systematic analysis for the Global Burden of Disease Study 2013. Lancet.

[3] US Department of Health and Human Services Preventing Tobacco Use among Youth and Young Adults: A Report of the Surgeon General 2012. http://www.surgeongeneral.gov/library/reports/preventing-youth-tobacco-use/.

[4] Pierce J.P., Choi W.S., Gilpin E.A., Farkas A.J., Merritt R.K. (1996). Validation of susceptibility as a predictor of which adolescents take up smoking in the United States. Health Psychol..

[5] Choi W.S., Gilpin E.A., Farkas A.J., Pierce J.P. (2001). Determining the probability of future smoking among adolescents. Addiction.

[6] Wakefield M., Kloska D.D., O’Malley P.M., Johnston L.D., Chaloupka F., Pierce J., Giovino G., Ruel E., Flay B.R. (2004). The role of smoking intentions in predicting future smoking among youth: Findings from Monitoring the Future data. Addiction.

[7] Azagba S., Asbridge M. (2013). School connectedness and susceptibility to smoking among adolescents in Canada. Nicotine Tob. Res..

[8] Veeranki S.P., Mamudu H.M., Anderson J.L., Zheng S. (2014). Worldwide never-smoking youth susceptibility to smoking. J. Adolesc. Health.

[9] Dube S.R., Arrazola R.A., Lee J., Engstrom M., Malarcher A. (2013). Pro-tobacco influences and susceptibility to smoking cigarettes among middle and high school students—United States, 2011. J. Adolesc. Health.

[10] Goniewicz M.L., Knysak J., Gawron M., Kosmider L., Sobczak A., Kurek J., Prokopowicz A., Jablonska-Czapla M., Rosik-Dulewska C., Havel C. (2014). Levels of selected carcinogens and toxicants in vapour from electronic cigarettes. Tob. Control.

[11] Bullen C., Howe C., Laugesen M., McRobbie H., Parag V., Williman J., Walker N. (2013). Electronic cigarettes for smoking cessation: A randomised controlled trial. Lancet.

[12] Grana R., Benowitz N., Glantz S.A. (2014). E-cigarettes: A scientific review. Circulation.

[13] Adkison S.E., O’Connor R.J., Bansal-Travers M., Hyland A., Borland R., Yong H.H., Cummings K.M., McNeill A., Thrasher J.F., Hammond D. (2013). Electronic nicotine delivery systems: International tobacco control four-country survey. Am. J. Prev. Med..

[14] Manzoli L., Flacco M.E., Fiore M., La Vecchia C., Marzuillo C., Gualano M.R., Ligurori G., Cicolini G., Capasso L., D’Amario C. (2015). Electronic Cigarettes Efficacy and Safety at 12 Months: Cohort Study. PLoS ONE.

[15] Gravely S., Fong G.T., Cummings K.M., Yan M., Quah A.C., Borland R., Yong H.H., Hitchman S.C., Mc Neill A., Hammond D. (2014). Awareness, trial, and current use of electronic cigarettes in 10 countries: Findings from the ITC project. Int. J. Environ. Res. Public Health.

[16] McMillen R.C., Gottlieb M.A., Shaefer R.M., Winickoff J.P., Klein J.D. (2015). Trends in Electronic Cigarette Use among U.S. Adults: Use is Increasing in Both Smokers and Nonsmokers. Nicotine Tob. Res..

[17] Agaku I.T., King B.A., Husten C.G., Bunnell R., Ambrose B.K., Hu S.S., Holder-Hayes E., Day H.R. (2014). Center for Disease Control and Prevention (CDC). Tobacco product use among adults–United States, 2012–2013. MMWR Morb. Mortal Wkly. Rep..

[18] Reid J.L., Rynard V.L., Czoli C.D., Hammond D. (2015). Who is using e-cigarettes in Canada? Nationally representative data on the prevalence of e-cigarette use among Canadians. Prev. Med..

[19] Carroll Chapman S.L., Wu L.T. (2014). E-cigarette prevalence and correlates of use among adolescents *versus* adults: A review and comparison. J. Psychiatr. Res..

[20] Dutra L.M., Glantz S.A. (2014). Electronic cigarettes and conventional cigarette use among U.S. adolescents: A cross-sectional study. JAMA Pediatr..

[21] Leventhal A.M., Strong D.R., Kirkpatrick M.G., Unger J.B., Sussman S., Riggs N.R., Stone M.D., Khoddam R., Samet J.M., Audrain-McGoven J. (2015). Association of Electronic Cigarette Use With Initiation of Combustible Tobacco Product Smoking in Early Adolescence. JAMA.

[22] Bunnell R.E., Agaku I.T., Arrazola R.A., Apelberg B.J., Caraballo R.S., Corey C.G., Coleman B.N., Dube S.R., King B.A. (2015). Intentions to smoke cigarettes among never-smoking US middle and high school electronic cigarette users: National Youth Tobacco Survey, 2011–2013. Nicotine Tob. Res..

[23] Coleman B.N., Apelberg B.J., Ambrose B.K., Green K.M., Choiniere C.J., Bunnell R., King B.A. (2015). Association between electronic cigarette use and openness to cigarette smoking among US young adults. Nicotine Tob. Res..

[24] Wang M.P., Ho S.Y., Leung L.T., Lam T.H. (2015). Electronic cigarette use and its association with smoking in Hong Kong Chinese adolescents. Addict. Behav..

[25] Moore G.F., Littlecott H.J., Moore L., Ahmed N., Holliday J. (2014). E-cigarette use and intentions to smoke among 10–11-year-old never-smokers in Wales. Tob. Control.

[26] Wills T.A., Sargent J.D., Knight R., Pagano I., Gibbons F.X. (2015). E-cigarette use and willingness to smoke: A sample of adolescent non-smokers. Tob. Control.

[27] Primack B.A., Soneji S., Stoolmiller M., Fine M.J., Sargent J.D. (2015). Progression to Traditional Cigarette Smoking After Electronic Cigarette Use Among US Adolescents and Young Adults. JAMA Pediatr..

[28] Stroup D.F., Berilin J.A., Morton S.C., Olkin I., Williamson G.D., Rennie D., Moher D., Becker B.J., Sipe T.A., Thacker S.B. (2000). Meta-analysis of observational studies in epidemiology: A proposal for reporting. Meta-analysis of Observational Studies in Epidemiology (MOOSE) group. JAMA.

[29] Higgins J.P., Thompson S.G., Deeks J.J., Altman D.G. (2013). Measuring inconsistency in meta-analyses. BMJ.

[30] DerSimonian R., Laird N. (1986). Meta-analysis in clinical trials. Control. Clin. Trials.

[31] Fairchild A.L., Bayer R., Colgrove J. (2014). The renormalization of smoking? E-cigarettes and the tobacco “endgame”. N. Engl. J. Med..

[32] Grana R.A. (2013). Electronic cigarettes: A new nicotine gateway?. J. Adolesc. Health.

[33] Zhan W., Dierker L.C., Rose J.S., Selya A., Mermelstein R.J. (2012). The natural course of nicotine dependence symptoms among adolescent smokers. Nicotine Tob. Res.

[34] Durmowicz E.L. (2014). The impact of electronic cigarettes on the paediatric population. Tob. Control.

[35] Ramôa C.P., Hiler M.M., Spindle T.R., Lopez A.A., Karaoghlanian N., Lipato T., Breland A.B., Shihadeh A., Eissenberg T. (2015). Electronic cigarette nicotine delivery can exceed that of combustible cigarettes: A preliminary report. Tob. Control.

[36] Farsalinos K.E., Spyrou A., Tsimopoulou K., Stefopoulos C., Romagna G., Voudris V. (2014). Nicotine absorption from electronic cigarette use: Comparison between first and new-generation devices. Sci. Rep..

[37] Okoli C.T., Kodet J. (2015). A systematic review of secondhand tobacco smoke exposure and smoking behaviors: Smoking status, susceptibility, initiation, dependence, and cessation. Addict. Behav..

[38] Czogala J., Goniewicz M.L., Fidelus B., Zielinska-Danch W., Travers M.J., Sobczak A. (2014). Secondhand exposure to vapors from electronic cigarettes. Nicotine Tob. Res..

[39] McAuley T.R., Hopke P.K., Zhao J., Babaian S. (2012). Comparison of the effects of e-cigarette vapor and cigarette smoke on indoor air quality. Inhal. Toxicol..

[40] Veeranki S.P., Mamudu H.M., Zheng S., John R.M., Cao Y., Kioko D., Anderson J., Ouma A.E. (2015). Secondhand smoke exposure among never-smoking youth in 168 countries. J. Adolesc. Health.

[41] Choi K., Forster J. (2013). Characteristics associated with awareness, perceptions, and use of electronic nicotine delivery systems among young US Midwestern adults. Am. J. Public Health.

[42] Leonardi-Bee J., Jere M.L., Britton J. (2011). Exposure to parental and sibling smoking and the risk of smoking uptake in childhood and adolescence: A systematic review and meta-analysis. Thorax.

